# Novel Library of Selenocompounds as Kinase Modulators

**DOI:** 10.3390/molecules16086349

**Published:** 2011-07-27

**Authors:** Daniel Plano, Elena Ibáñez, Alfonso Calvo, Juan Antonio Palop, Carmen Sanmartín

**Affiliations:** 1 Department of Organic and Pharmaceutical Chemistry, University of Navarra, Irunlarrea, 1, Pamplona E-31008, Spain; 2 Oncology Division, Center for Applied Medical Research, CIMA, University of Navarra, Pío XII, 53, Pamplona E-31008, Spain

**Keywords:** selenium, kinases, PI3K, AKT, mTOR

## Abstract

Although the causes of cancer lie in mutations or epigenic changes at the genetic level, their molecular manifestation is the dysfunction of biochemical pathways at the protein level. The 518 protein kinases encoded by the human genome play a central role in various diseases, a fact that has encouraged extensive investigations on their biological function and three dimensional structures. Selenium (Se) is an important nutritional trace element involved in different physiological functions with antioxidative, antitumoral and chemopreventive properties. The mechanisms of action for selenocompounds as anticancer agents are not fully understood, but kinase modulation seems to be a possible pathway. Various organosulfur compounds have shown antitumoral and kinase inhibition effects but, in many cases, the replacement of sulfur by selenium improves the antitumoral effect of compounds. Although Se atom possesses a larger atomic volume and nucleophilic character than sulfur, Se can also formed interactions with aminoacids of the catalytic centers of proteins. So, we propose a novel chemical library that includes organoselenium compounds as kinase modulators. In this study thirteen selenocompounds have been evaluated at a concentration of 3 or 10 µM in a 24 kinase panel using a Caliper LabChip 3000 Drug Discover Platform. Several receptor (EGFR, IGFR1, FGFR1…) and non-receptor (Abl) kinases have been selected, as well as serine/threonine/lipid kinases (AurA, Akt, CDKs, MAPKs…) implicated in main cancer pathways: cell cycle regulation, signal transduction, angiogenesis regulation among them. The obtained results showed that two compounds presented inhibition values higher than 50% in at least four kinases and seven derivatives selectively inhibited one or two kinases. Furthermore, three compounds selectively activated IGF-1R kinase with values ranging from −98% to −211%. In conclusion, we propose that the replacement of sulfur by selenium seems to be a potential and useful strategy in the search of novel chemical compound libraries against cancer as kinase modulators.

## 1. Introduction

**Figure 1:**
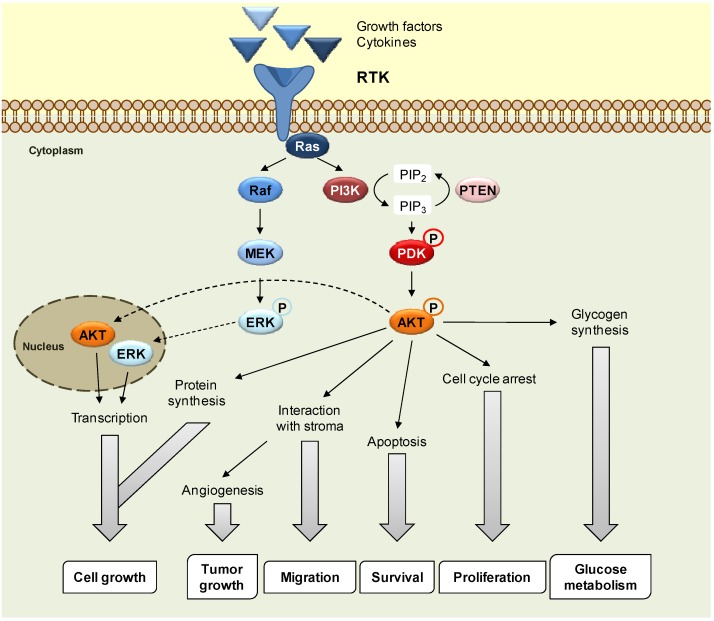
Schematic overview of the key cellular signaling pathways that are commonly affected by oncogenic alterations and may be targets for therapy.

Nowadays, malignancies are still crucial problems in the healthcare system in many aspects which include efficacy of therapeutic modalities, quality of patients’ life and cost of treatments. Protein kinase inhibitors have been validated for a number of diseases, including cancer. Thus, there is ongoing interest in the discovery of specific and selective protein kinase inhibitors for the development of new drugs, and for their use as pharmacological tools, which represent an emerging challenge in drug discovery [1]. Irreversible inhibitors bind to kinase active site in a covalent and irreversible form, most frequently by reacting with a nucleophilic cysteine residue, located near the ATP binding pocket [2,3]. Deregulation of kinase activity has emerged as a major mechanism through which cancer cells escape from normal physiological restrictions over growth and survival, leading to cell proliferation. There are more than 518 kinases, which are grouped into ~20 known families based on structural relatedness [4]. In general, low molecular weight inhibitors [5] are the most commonly used agents to target protein kinases in cancer treatment. Very few drugs are truly selective for a single target. In fact, most biologically active small molecules have a degree of promiscuity by their nature. For this reason, the identification of multikinase inhibitors with specific multiple activity profiles is currently an area of great interest in the pharmaceutical industry, especially for the treatment of cancer [6]. It is known that several protein kinase pathways play essential roles in mediating mitogenic and antiapoptotic signals and can regulate cell proliferation and survival [7]. At present it is difficult, if not impossible, to consider a wide kinase panel to describe a multikinase inhibition route that encompasses all the connections and combinations that are possibly implicated in cancer modulation [8]. Among the promising targeted therapies for cancer treatment, phosphatidylinositol 3-kinase pathway (PI3K) inhibitors have in the last 3 years retained the attention of both academic institutions and pharmaceutical companies. The PI3K signaling pathway is crucial to many aspects of cell growth and survival [9] (Figure 1). Activation of the PI3K pathway results in a disturbance of control of cell growth and survival, which contributes to a competitive growth advantage, metastatic competence and, frequently, therapy resistance. This pathway is therefore an attractive target for the development of novel anticancer agents [10]. AKT, also termed protein kinase B (PKB), is the most crucial proximal node downstream of the receptor tyrosine kinase (RTK)-PI3K complex, which mediates a broad spectrum of cellular functions, ranging from control of cell proliferation and survival to modulation of intermediary metabolism, which makes AKT an attractive therapeutic target.

The presence of Ras and Raf mutations in cancer highlights the importance of the mitogen-activated protein kinase (MAPK) pathway as a therapeutic approach [11]. Ras has been particularly difficult to target because its enzymatic activity is rather low and depends on association with the GTPase activating proteins to stimulate its inactivation. The extracellular-signal-regulated kinase (ERK) has also proven to be a difficult target, due to the difficulties inherent in making ERK-specific drugs. Mitogen-activated protein kinase kinases 1 and 2 (MEK1 and MEK2) are closely related dual-specificity kinases, capable of phosphorylating both serine/threonine and tyrosine residues of their substrates ERK1 and ERK2. They are the only known catalytic substrates of Raf kinases. The fact that ERK is the only known substrate of MEK, when coupled with the observation that ERK is commonly activated in both tumour cell lines and patient tumors, has fueled strong interest in developing pharmacological inhibitors of MEK as a means to block ERK activation [12].

**Figure 2:**
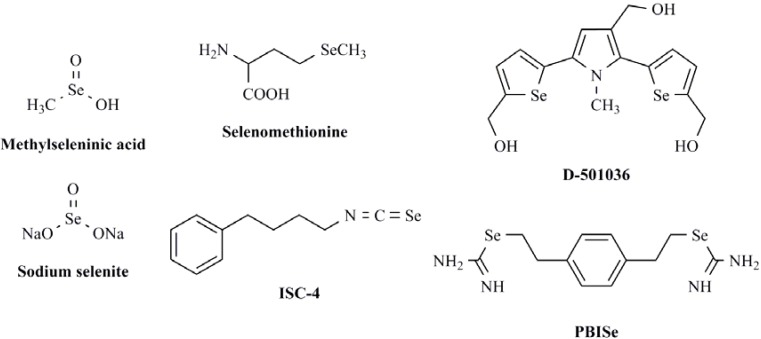
Chemical structures of some selenocompounds with kinase activity.

Several reports have recently highlighted that selenocompounds are able to induce tumor cell apoptosis through distinct mechanisms according to cell type and compound pattern. These mechanisms include survival pathways modulation, among which the PI3K/AKT pathway appears as a common target for the selenocompounds studied [13,14,15,16,17,18,19,20,21,22,23,24,25,26]. It is remarkable that they can modulate different kinases at the same time and their effectiveness varies depending on the genetic background of the tumor cells [27,28,29,30,31]. The selenocompounds recognized as kinase modulators (Figure 2) include selenomethyl derivatives such as methylseleninic acid [13,14,25] and selenomethionine [15,16,23,27] that act alone or as methylselenol precursors [28,29]; heterocycles containing a selenium atom in the ring (D-501036) [32] and compounds with a selenourea (PBISe) [21] and isoselenocyanate (ISC-4) [24,25,26,33] moieties. In addition, molecular symmetry is commonly presented in these structures [17,18,19,20,21].

Kinase-directed libraries have proven to be useful in pharmacological kinase studies and for hit generation in drug discovery. Taking into account the urgent need of new chemical approaches for the discovery of novel compounds that are capable of inhibiting kinase activation, we describe in this paper the screening of a kinase-directed library, which comprises a broad structural variety and includes: *Se*-alkyl imidoselenocarbamates (**A**), selenylacetic acids (**B**), selenodiazoles (**C**), selenolderivatives (**D**) and a diselenide compound (**E**). This approach is based on the promising cytotoxic activity of compounds previously reported by our group [34,35,36,37,38] and will be considered, as a starting point for a new strategy, in the design and development of new anticancer candidates.

## 2. Results and Discussion

### 2.1. Chemistry

**Figure 3:**
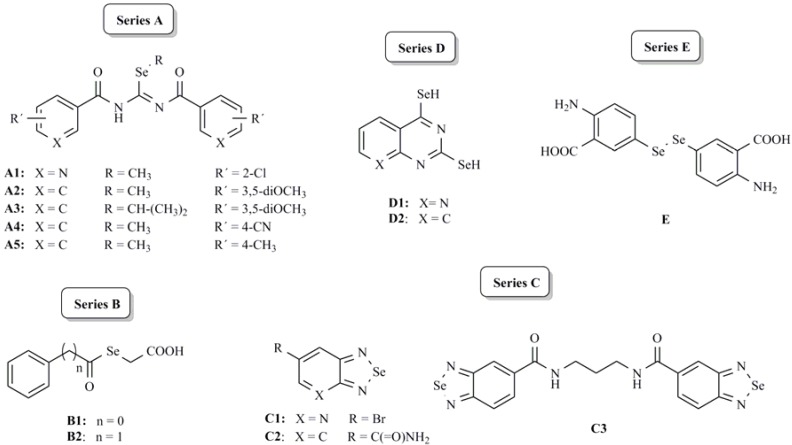
General structures for studied compounds.

The compounds reported in this paper have been grouped into five series **A**–**E** as illustrated in Figure 3.

The *Se*-alkyl imidoselenocarbamates **A** were synthesized following our previously published methods [34], starting from the appropriate *Se*-alkyl selenourea hydroiodide and the corresponding acyl chloride. The preparation of the compounds of **B** series was carried out by reaction of acyl chlorides with sodium hydrogen selenide followed by reaction with alpha-bromoacetic acid [35]. Compounds of **C** series were synthesized from the appropriate *ortho*-aromatic diamine and selenium dioxide [36] and transformed into amides by reaction of the carboxylic acid with thionyl chloride and the appropriate amine or diamine. Compounds of **D** series were prepared by reaction of the corresponding quinazoline or pyrido[2,3-*d*]pyrimidine with phosphoryl chloride and the intermediate dihalogenated derivative was treated with selenourea in absolute ethanol [37]. Finally, the diselenide compound **E** was synthesized by addition of selenium dioxide to malononitrile in DMSO followed by reaction with *ortho*-amino benzoic acid. The intermediate selenocyanate was reduced to diselenide with sodium borohydride [38,39].

### 2.2. Biological Evaluation

#### 2.2.1. Kinase screening

The thirteen compounds described in this paper have been evaluated against 24 cancer-relevant protein kinases belonging to different families of the kinome in order to reveal their potential therapeutic activity as kinase inhibitors (Table 1). The compounds were tested at a single concentration of 3 or 10 µM (concentration varied depending on the assay) using a Caliper LabChip 3000 Drug Discover Platform. The experiments were performed in duplicate. Details of the experiment are provided in the experimental section. Two major questions needed to be tackled. Firstly, could these compounds act as kinase inhibitors? And if they can, which kinases are affected? Compounds **A1–A5**, **B1–B2**, **C1–C3**, **D1–D2** and **E** were selected as they represent the five major structural patterns of our design and were cytostatic or cytotoxic in PC-3 prostate cancer cell line. The screening showed that many of the compounds tested did not have kinase inhibitory activity. It should be taken into account that a panel of 24 kinases is slightly limited for kinome selectivity studies, and therefore the results should be interpreted cautiously. Nevertheless, the data from these experiments showed interesting differences in kinase inhibition between structural subsets within the library. In Table 1, where compounds have been grouped based on their structure, it can be highlighted that symmetric compounds of series **A** with an imidoselenocarbamate moiety inhibited an important proportion of the tested kinases, most notably derivatives **A2** and **A4**. The inhibition was above 61% and 81% in Abl kinase, respectively, a fusion tyrosine kinase protein which is responsible for 90% of chronic myeloid leukemia (CML) cases. AKT1 a serine/threonine kinase, which is over-expressed in a large number of tumors including breast, prostate, lung, pancreas, liver, ovary and colorectum was also inhibited (above 71% and 89% inhibition, respectively). In other kinases, the inhibition percentages caused by compounds **A2** and **A4** were between 40% and 73%. These kinases include CDK2, CDK9, CKIT, GSK3a, MAPK3, ErbB4. CDK1 and CDK2 are cyclin-dependent kinases (CDKs), cell cycle regulators which are appealing targets for multiple myeloma (MM) therapy given the increased proliferative rates of tumour cells in advanced versus early stages of MM. CKIT is the receptor for the stem cell growth factor (SCF) and belongs to the platelet-derived growth factor receptor (PDGFR) family. It plays an essential tumor-cell-intrinsic role in many types of cancer, either providing the tumorigenic force when aberrantly activated or conferring stem-like features characterizing the most aggressive variants [40]. CKIT-targeted therapy with tyrosine kinase inhibitors may ideally work against both tumor and stromal cells. These derivatives were able also to inhibit other oncogenic kinases, as showed in Table 1, but the inhibition percentages were below 40%.

Selenylacetic acids (**B1**, **B2**) and selenodiazoles did not significantly inhibit kinase activity, with the exception of **C3**, that caused an inhibition of 54% and 44% in CK1A and GSK3a, respectively. This compound, as well as compounds **A1**–**A5**, possesses molecular symmetry, a property frequently present in kinase modulators and anticancer drugs [41,42,43,44]. Quinazoline (**D1**) and pyrido[2,3-*d*]pyrimidine (**D2**) derivatives slightly inhibited Aurora A (AurA), but caused a strong inhibition of ErbB4 kinase activity. Aurora A and Aurora B (AurB) are essential components of the mitotic pathway, ensuring the appropriate chromosome assembly, the formation of the mitotic spindle and the cytokinesis. Overexpression of AurA and AurB has been observed in several tumor types and has been linked to a poor prognosis in cancer patients. Quinazolines have previously been described as Aurora kinase inhibitors [45,46]. On the other hand, ErbB4, a receptor tyrosine kinase which is a member of the epidermal growth factor receptor subfamily has also been reported to be modulated by quinazoline [47] as well as by pyridopyrimidines [48]. Finally, compound **E**, a symmetrical diselenide derivative, mildly inhibited cyclin kinase activity.

It should be underlined that three compounds (**C1**, **D1** and **D2**) are potent activators of insulin-like growth factor 1 receptor (IGF-1R) whose pathway plays a major role in cancer growth, tumor cell survival and resistance to therapy. Activation of IGF-1R by IGF-1 or IGF-2 leads to activation of both the MAPK and PI3K/AKT/mTOR pathways, promoting proliferation and inhibiting apoptosis [49]. IGF-1R activation could represent a novel therapeutic approach since it has been previously recognized as a key pathway involved in the mechanisms by which a gastrointestinal neuropeptide, named neurotensin, modulates inflammation [50] or regulates immune responses [51] among others functions. For these reasons the derivatives **C1**, **D1** and **D2** could be considered as promising candidates in several diseases.

Data obtained in our study suggest the imidoselenocarbamates **A2** and **A4**, which have proven to be multi-kinase inhibitors, as possible leaders of these series. In order to validate their potential therapeutic use, their *in vitro* activities have been further pursued.

**Table 1:** Summary of kinase screening (% inhibition at 3 or 10 μM): Darker colour indicates greater % inhibition.

Kinases	Compounds												
	A1	A2	A3	A4	A5	B1	B2	C1	C2	C3	D1	D2	E
**Abl ** **^a^**	-4 ± 3	61 ± 3	33 ± 6	82 ± 7	21 ± 8	4 ± 1	5 ± 10	10 ± 10	8 ± 5	9 ± 14	40 ± 4	25 ± 9	19 ± 14
**AKT1 ^a^**	2 ± 2	71 ± 8	36 ± 9	89 ± 3	5 ± 1	-12 ± 5	-6 ± 5	12 ± 13	15 ± 12	13 ± 10	38 ± 12	9 ± 4	11 ± 2
**AurA ^a^**	74 ± 3	5 ± 2	-5 ± 2	13 ± 12	21 ± 7	17 ± 3	12 ± 6	3 ± 3	5 ± 1	-7 ± 15	46 ± 4	42 ± 6	21 ± 7
**CDK2 ^a^**	-3 ± 5	64 ± 16	32 ± 11	73 ± 6	20 ± 9	-2 ± 1	-4 ± 2	26 ± 16	6 ± 1	3 ± 7	25 ± 5	8 ± 6	3 ± 6
**CDK9 ^a^**	19 ± 2	45 ± 1	5 ± 6	24 ± 6	21 ± 3	17 ± 1	16 ± 4	6 ± 2	24 ± 4	17 ± 9	16 ± 2	14 ± 7	54 ± 2
**CK1A ^b^**	18 ± 15	16 ± 11	21 ± 14	16 ± 19	13 ± 5	11 ± 1	15 ± 4	15 ± 6	17 ± 2	54 ± 1	12 ± 1	10 ± 1	12 ± 1
**CK2 ^b^**	4 ± 1	19 ± 6	12 ± 3	6 ± 7	0 ± 1	-1 ± 2	9 ± 5	18 ± 5	20 ± 3	15 ± 3	17 ± 3	9 ± 3	3 ± 3
**CKIT ^a^**	-15 ± 3	64 ± 15	-1 ± 11	58 ± 8	3 ± 10	1 ± 14	-1 ± 3	13 ± 13	-15 ± 8	-10 ± 5	3 ± 8	18 ± 15	-5 ± 4
**CRAF ^a^**	-8 ± 2	-6 ± 1	-6 ± 1	-6 ± 3	-7 ± 1	1 ± 3	0 ± 2	1 ± 4	2 ± 1	-1 ± 1	2 ± 1	15 ± 9	-10 ± 4
**EGFR ^a^**	-4 ± 9	7 ± 6	0 ± 3	11 ± 7	1 ± 1	-4 ± 5	1 ± 2	−2 ± 5	-4 ± 2	-4 ± 5	3 ± 5	2 ± 2	-2 ± 4
**ErbB4 ^a^**	24 ± 3	24 ± 12	43 ± 9	15 ± 10	29 ± 4	30 ± 1	29 ± 1	34 ± 5	32 ± 6	22 ± 12	65 ± 4	70 ± 2	35 ± 2
**FAK2 ^a^**	4 ± 1	0 ± 4	1 ± 2	5 ± 2	-1 ± 3	5 ± 1	5 ± 1	2 ± 1	4 ± 2	6 ± 2	8 ± 4	5 ± 1	3 ± 1
**FGFR1 ^a^**	-6 ± 2	1 ± 1	-9 ± 6	3 ± 3	6 ± 2	7 ± 2	-2 ± 1	-2 ± 2	1 ± 1	-3 ± 10	2 ± 3	3 ± 2	-2 ± 2
**GSK3a ^b^**	8 ± 2	50 ± 7	41 ± 3	42 ± 10	22 ± 9	23 ± 8	28 ± 4	37 ± 2	31 ± 8	44 ± 4	34 ± 6	37 ± 10	46 ± 13
**IGF1R ^a^**	-2 ± 4	-2 ± 4	3 ± 2	-18 ± 4	-4 ± 4	-9 ± 2	-2 ± 7	-98 ± 9	2 ± 4	-5 ± 4	-211 ± 6	−161 ± 2	4 ± 4
**JNK2 ^b^**	2 ± 3	9 ± 5	-1 ± 9	6 ± 3	4 ± 2	1 ± 6	4 ± 3	6 ± 2	6 ± 6	5 ± 6	8 ± 1	3 ± 5	6 ± 4
**KDR ^a^**	1 ± 3	16 ± 2	6 ± 4	33 ± 5	3 ± 3	-1 ± 1	4 ± 1	4 ± 1	11 ± 1	2 ± 1	14 ± 6	1 ± 4	4 ± 1
**MAPK1 ^a^**	-15 ± 2	-3 ± 1	-3 ± 2	21 ± 1	-4 ± 1	-7 ± 1	-11 ± 3	-4 ± 1	2 ± 3	2 ± 5	3 ± 4	-4 ± 1	-35 ± 2
**MAPK11 ^b^**	-4 ± 5	8 ± 7	3 ± 6	-8 ± 8	0 ± 1	-8 ± 11	-8 ± 2	-6 ± 7	0 ± 1	-7 ± 1	1 ± 4	0 ± 10	2 ± 5
**MAPK14 ^b^**	1 ± 3	17 ± 5	11 ± 1	14 ± 3	-2 ± 7	3 ± 4	7 ± 4	9 ± 2	15 ± 4	13 ± 6	4 ± 7	11 ± 4	18 ± 2
**MAPK3 ^a^**	-16 ± 5	51 ± 1	18 ± 8	13 ± 20	14 ± 13	-6 ± 4	-8 ± 10	-6 ± 11	-6 ± 2	-8 ± 6	10 ± 3	2 ± 4	-8 ± 11
**PDGFRb ^a^**	-14 ± 9	18 ± 6	-3 ± 2	-8 ± 6	4 ± 1	5 ± 2	21 ± 8	-32 ± 15	-5 ± 1	-8 ± 2	-11 ± 10	-34 ± 3	31 ± 3
**PKA ^b^**	0 ± 1	2 ± 1	3 ± 1	0 ± 4	0 ± 1	1 ± 1	3 ± 2	7 ± 6	5 ± 1	4 ± 3	5 ± 2	2 ± 2	5 ± 1
**PKCA ^a^**	18 ± 8	-3 ± 6	2 ± 4	-16 ± 3	-8 ± 2	25 ± 4	-1 ± 1	-6 ± 4	11 ± 3	9 ± 9	-2 ± 19	-18 ± 13	-10 ± 4

^a^ Compounds tested at 10 μM; ^b^ Compounds tested at 3 μM.

#### 2.2.2. Effect of compounds **A2** and **A4** on PI3K/AKT and MAPK pathways

**Figure 4:**
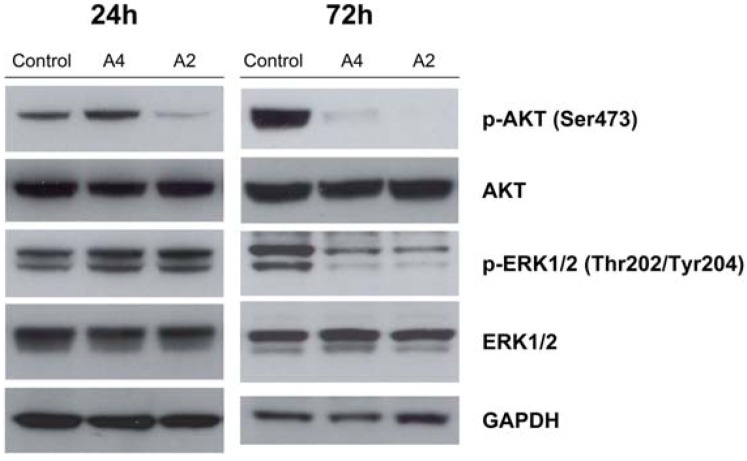
Intracellular signaling events induced by compounds **A2** and **A4** *in vitro*. PC-3 human prostate cancer cells were treated with 10 μM of compounds **A2** and **A4** for 24 and 72h. Cells were lysed for Western Blot analysis using antibodies specific to p-AKT, AKT, p-ERK, ERK and GAPDH.

Because the PI3K/AKT pathway is highly relevant in solid tumors, and AKT activity has been seriously inhibited by these compounds, we focused our experiments in the blockade of this signaling cascade. The MAPK signaling inhibition has also been studied because the cross-talk between these two pathways has been widely described [52,53]. Human PC-3 prostate cancer cells have been chosen for these studies based on previously reported *in vitro* growth inhibition studies, which showed that compounds **A2** and **A4** effectively blocked proliferation in PC-3 [34]. This cell line is PTEN-deficient and exhibits high phosphorylation of Ser473 on AKT, suggesting an overactive AKT pathway [54]. In addition, many of the kinase inhibitory effects caused by other selenocompounds have been observed in prostate cancer cells [13,15,16,18,19,23]. Two h treatment with 10 μM of compound **A2** inhibited AKT phosphorylation at Ser473 and ERK phosphorylation at Thr202/Tyr204, two crucial proximal nodes of the PI3K and MAPK pathways, respectively. However, it was necessary to increase the incubation time to 72 hours to obtain the same inhibitory effect by compound **A4** in the same cell line (Figure 4). Total AKT and ERK signals remained constant throughout the treatment.

#### 2.2.3. mTOR signaling alteration by compounds **A2** and **A4**

One of the major signal coordinators in mammalian cells, mTOR, is commonly involved in cancer [55]. This serine/threonine protein kinase functions as a point of convergence of multiple upstream inputs, including receptor-mediated external mitogenic signals and information on the nutritional/energetic state of the cell. One of the main positive regulators of mTOR is the PI3K/AKT pathway. mTOR receives these multiple signals and integrates them, through mechanisms by which it engages in two distinct multicomplexes, termed mTORC1 and mTORC2, and in turn it controls several cellular functions, such as protein synthesis, transcriptional processes, cell growth, apoptosis, and release of angiogenic factors [56]. In particular, mTOR plays a major role in promoting cell survival by downregulating apoptosis. AKT is phosphorylated at Ser473 by mTORC2 whereas mTORC1 is a downstream substrate of AKT. Although no mutations in mTOR have been found in cancer, it is frequently hyperactive in tumour cells due to sustained mitogenic stimulation, particularly through the PI3K pathway, most often as a result of lowered PTEN function.

**Figure 5:**
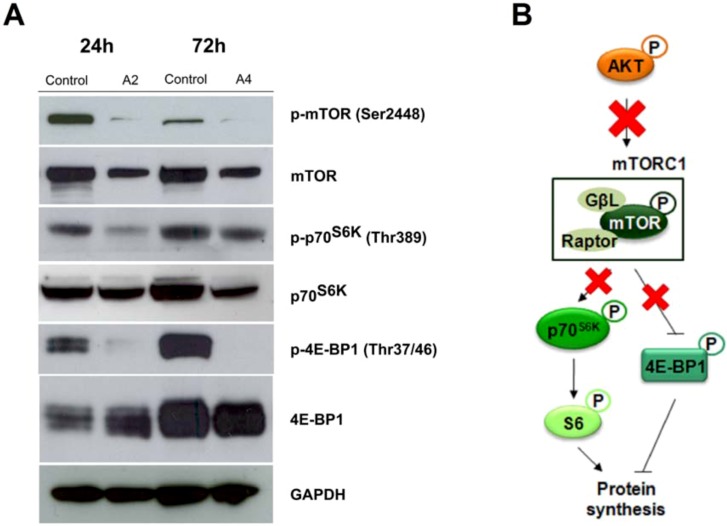
(**A**) PC-3 human prostate cancer cells were treated with 10 μM of compound **A4** for 24 and **A2** for 72h. Cells were lysed for Western Blot analysis by using antibodies specific to p-mTOR, mTOR, p-p70^S6K^, p70^S6K^, p-4E-BP1, 4E-BP1 and GAPDH; (**B**) scheme of the intracellular signaling blockade induced by compounds **A2** and **A4** *in vitro*.

With the aim of investigating the PI3K/AKT/mTOR pathway alteration, the modulation of the downstream substrate of AKT, mTORC1 has been studied under compound **A2** and **A4** exposure (Figure 5A). Both compounds inhibited mTOR phosphorylation at Ser2448, this inhibition being again observed at different incubation times. mTORC1 serves as a molecular sensor to control protein synthesis via regulation of two separate downstream targets, the 70 kDa S6 kinase (p70^S6K^) and the eukaryotic initiation factor 4E binding protein-1 (4E-BP1) [57] (Figure 5B), which are also dephosphorylated by compound **A2** and **A4** in PC-3 cells. As expected, total protein signals remained constant throughout the treatment.

## 3. Experimental

### 3.1. Synthetic Chemistry and Compound Characterization

Compounds were prepared using the synthetic routes previously reported [34,35,36,37,38,39]. All library compounds were evaluated to verify identity by IR, ^1^H-NMR and to verify purity by elemental microanalyses. The ^1^H-NMR spectra were recorded on a Bruker 400 Ultrashield^TM^ spectrometer (Rheinstetten, Germany) using TMS as the internal standard. The IR spectra were obtained on a Thermo Nicolet FT-IR Nexus spectrophotometer with KBr pellets. Elemental microanalyses were carried out on vacuum-dried samples using a LECO CHN-900 Elemental Analyzer.

### 3.2. Kinase Inhibition Screening Assay

Compounds were tested by microfluidic mobility shift assay [58] using a LabChip_ EZReader II from Caliper Life Sciences and ProfilerPro kinase selectivity assay kits from the same supplier. Buffers were added using a Thermo Multidrop Combi and all other liquid transfers were carried out with a PerkinElmer Evolution P3 robotic liquid handling system to eliminate variation due to the time needed to transfer all reagents. Test compounds were dissolved in DMSO to give 1 mM solutions which were subsequently diluted to give 3 and 10 μM under final assay conditions. Appropriate fluorescently tagged peptides were used for each kinase and assays were run at apparent Km ATP for each protein. Positive controls contained no inhibitor and negative controls contained no ATP. Phosphorylated and unphosphorylated peptides were separated by electrophoresis and the percentage conversion was calculated as a ratio of the area under the phosphorylated peptide peak relative to the sum of both peaks. Exact conditions for each experiment are listed in Supplementary Table S1. Enzymes tested were as follows: Abl, AKT1, AurA, CDK2/cycA, CDK9, CK1α, CK2, cKIT, c-RAF, EGFR, ErbB4, FAK2, FGFR1, GSK3α, IGF1R, JNK2α2, KDR, MAPK1, MAPK3, MAPK11, MAPK14, PDGFRβ, PKA, PKCα. Staurosporine, 5-Iodotubercidin, SB203580, K252a (Calbiochem) were used as positive controls to allow comparisons between plates.

### 3.3. Cell Lines and Cell Culture

Cell culture materials were obtained from BD Bioscience. The immortalized cell line PC-3 (prostate carcinoma) was purchased from the American Type Culture Collection (ATCC). PC-3 cells were cultured in RPMI 1640 medium (Lonza) containing heat-inactivated 10% fetal bovine serum (Hyclone) and 1% penicillin/streptomycin (Gibco) in a humidified incubator at 37 °C in 5% CO_2_. For Western Blot analysis, cells were plated at 80% confluence and treated with the selenocompound for 24 and 72 h at a concentration of 10 μM or with DMSO (0.2%, untreated control).

### 3.4. Western Blot Analysis

After a 24-hours of drug-exposure, cells were lysed in cell lysis buffer consisting of: 20 mmol/L Tris-HCl (pH 7.5), 150 mmol/L NaCl, 1% Triton X-100, supplemented with protease inhibitors (Roche) and phosphatase inhibitors (10 mmol/L sodium fluoride and 10 mmol/L sodium orthovanadate, Sigma) for 30 min on ice. Lysates were centrifuged at 13,200 × g for 15 min at 4 °C to remove cell debris. The non-protein fraction and supernatants were stored at −80 °C before use. Protein concentration was determined with the BCA Protein Assay (Pierce). Cell Samples (20–40 μg) were placed in SDS-sample buffer and 2% 2-β-mercaptoethanol, boiled for 5 min and subjected to SDS-PAGE on 12% Tris-glycine gels (Invitrogen). Separated proteins were transferred onto 0.22 µm nitrocellulose membranes (BIORAD) at 100V for 1 hand stained with Ponçeau solution. The membranes were incubated in blocking solution (5% non fat dry milk -TBS-Tween-20) for 1 h at room temperature. Primary specific antibodies were incubated in 5% milk-TBS-Tween-20 (1 h, room temperature) to detect pThr389 p70^S6K^, p70^S6K^, pThr37/46 4E-BP1, 4E-BP1 (all of them at 1:1,000 dilution, Cell Signaling), pSer473 AKT, AKT, pSer2448 mTOR, mTOR, pThr202/Tyr204 ERK1/2, ERK1/2 (all of them at 1:2000 dilution, Cell Signaling) and GAPDH (1:20,000 dilution, AbD Serotec). After incubation with the HRP-conjugated secondary antibody at 1:5,000 dilution (GE Healthcare) in 5% milk-TBS-Tween-20 (1 h, room temperature), a Lumi Light Plus chemoluminiscence kit (Roche) was used for visualization. Western blots are representative of at least three independent experiments.

## 4. Conclusions

A screening library which contained thirteen selenium derivatives was tested against a panel of human kinases and their inhibitory activity was shown. Some structure-inhibitory activity relationships could be discerned from this screening. From the results four types of behaviour can be highlighted for these series:

Behavior 1: Compounds that do not inhibit kinases significantly. The compounds are **A5**, **B1**, **B2**, **C1** and **C2**.
Behavior 2: Compounds that inhibit, with similar intensity, two kinases and activate one kinase. These compounds, which are representative of **D** series, are **D1** and **D2**, that caused Aur A and ErbB4 inhibition and IGF-1R activation.
Behavior 3: Compounds from different series which inhibited the same kinase activity. For instance, compounds **A3**, **C3** and **E** inhibited GSK3a; compounds **A1**, **D1** and **D2** AurA and compounds **A3**, **D1** and **D2** ErbB4.
Behavior 4: Multikinase inhibitor compounds, which include **A2** and **A4**.

Considering all of the biological results in PC-3 cell line previously reported by us as well as the kinase inhibitory activity profile, compounds A2 and A4 were found to be the most appropriate as candidates for further testing with the aim of finding out more selective and active anticancer drugs. In PC-3 cells, compounds A2 and A4 caused an important inhibitory effect in AKT and ERK phosphorylation, two key nodes in PI3K and MAPK pathways, respectively. Furthermore, consistent with its possible inhibitory action on both mTORC1 and mTORC2, compounds A2 and A4 also dephosphorylated p70^S6K^ and 4E-BP1 as well as AKT on Ser473 in PC-3 cells, emerging as promising inhibitors of the PI3K/AKT/mTOR and MAPK pathways. In spite of both of them inhibited Abl, a kinase linked to response to oxidative stress through interaction with glutathione peroxidase I (GPx1) [59], these compounds did not exhibited antioxidant-prooxidant activity according to the results reported previously by us [38]. Further mechanistic studies are necessary to determine whether the effects of compounds A2 and A4 on cell proliferation are only associated with these pathways inhibition or whether additional inhibitory activities are also involved. Available evidences from the literature have not provided a full understanding of selenium in tumorigenesis. Recently, a novel role of selenium has been proposed through ATM-kinase that plays a pivotal role in the DNA damage induced senescence response in a manner dependent on ROS especially at the interphase between the precancerous stages [60,61]. The study of these new targets may help to discover a potential therapeutic pathway for the control and prevention of cancer.

## Supplementary Materials

Supplementary File 1
