# Supplementary material for: Novel Library of Selenocompounds as Kinase Modulators
Source: Molecules. 2011 Jul 27;16(8):6349–64. doi: 10.3390/molecules16086349 (PMC6264252; doi:10.3390/molecules16086349)
Supplement: Supplementary File 1 [file molecules-16-06349-s001.pdf]

Table S1. Kinase inhibition screening assay conditions.

Kinase	Species	Reference compound	Cation	Buffer, pH	Detergent	ATP Km (μM)	Enzyme Concentration (μM)	Tested Compound Concentration (μM)	Peptide
Abl	Human	Staurosporine	Mg	Hepes, 7.5	Brij	14	0.7	10	FL-EAIYAAPFAKKK-$CONH_2$
AKT1	Human	Staurosporine	Mg	Hepes, 7.5	Brij	19	1.1	10	FL-GRPRTSSFAEG-$CONH_2$
AurA	Human	Staurosporine	Mg	Hepes, 7.5	Brij	25	4	10	FL-LRRASLG-$CONH_2$
CDK2	Human	Staurosporine	Mg	Hepes, 7.5	Brij	36	1.2	10	FL-QSPKKG-$CONH_2$
CDK9	Human	Staurosporine	Mg	MOPS, 6.0	CHAPS0	3	5	10	FITC-GSRTPMY
CK1α	Human	5-iodotubercidin	Mg	Hepes, 7.5	Brij	6.9	4.64	3	FL-KRRRALpSVASLPGL-$CONH_2$
CK2	Human	Staurosporine	Mg	Hepes, 7.5	Brij	3.95	20	3	FL-RRRDDDSDDD-$CONH_2$
cKIT	Human	Staurosporine	Mn	Hepes, 7.5	Brij	300	20	10	FL-EAIYAAPFAKKK-$CONH_2$
c-RAF	Human	SB203580	Mn	Hepes, 7.5	Brij	6.2	36	10	FL-GQLIDSMANSFVGTR-$CONH_2$
EGFR	Human	Staurosporine	Mn	Hepes, 7.5	Brij	2.5	20	10	FL-KKAEEEEYFEYFFLVAKK-$CONH_2$
ErbB4	Human	Staurosporine	Mn	Hepes, 7.5	Triton x-100	2	5	10	FITC-KKKKEAIYFFF-$CONH_2$
FAK2	Human	Staurosporine	Mg	Tricine, 8.0	Triton x-100	47.9	5.85	10	FITC-KKKKEAIYFFF-$CONH_2$
FGFR1	Human	Staurosporine	Mg	MOPS, 6.5	Triton x-100	506.67	6	10	FL-KKSRGDYMTMQIG-$CONH_2$
GSK3α	Human	Staurosporine	Mg	Hepes, 7.5	Brij	28.5	0.5	3	FL-KRREILSRRPpSYR-COOH
IGF1R	Human	Staurosporine	Mn	Hepes, 7.5	Brij	2930	20	10	FL-KKSRGDYMTMQIG-$CONH_2$
JNK2α2	Human	SB203580	Mg	Hepes, 7.5	Brij	35.5	24	3	FL-KRELVEPLTPSGEAPNQALLR-$CONH_2$
KDR	Human	Staurosporine	Mg	Hepes, 7.5	CHAPSO	450	2.5	10	FL-EEPLYWSFPAKKK-$CONH_2$
MAPK1	Human	K525a	Mg	Hepes, 7.5	Brij	14	1.35	10	FL-IPTSPITTTYFFFKKK-COOH
MAPK3	Human	Staurosporine	Mg	Hepes, 7.5	Brij	21	1.35	3	FL-IPTSPITTTYFFFKKK-COOH
MAPK11	Human	SB203580	Mg	Hepes, 7.5	Brij	107.33	7.5	3	FL-IPTSPITTTYFFFKKK-COOH
MAPK14	Human	SB203580	Mg	Hepes, 7.5	Brij	100	10	10	FL-IPTSPITTTYFFFKKK-COOH
PDGFRb	Human	Staurosporine	Mn	MOPS, 6.5	Triton x-100	12	50	10	FITC-Aha-KKKKKEEIYFFF-$CONH_2$
PKA	Human	Staurosporine	Mg	Hepes, 7.5	Brij	5	0.175	3	FL-LRRASLG-$CONH_2$
PKCA	Human	Staurosporine	Mg	Hepes, 7.5	Brij	23	0.475	10	FL-KRPSKRAKA-COOH
